# Celastrol mitigates inflammation in sepsis by inhibiting the PKM2-dependent Warburg effect

**DOI:** 10.1186/s40779-022-00381-4

**Published:** 2022-05-20

**Authors:** Piao Luo, Qian Zhang, Tian-Yu Zhong, Jia-Yun Chen, Jun-Zhe Zhang, Ya Tian, Liu-Hai Zheng, Fan Yang, Ling-Yun Dai, Chang Zou, Zhi-Jie Li, Jing-Hua Liu, Ji-Gang Wang

**Affiliations:** 1 Artemisinin Research Center, and Institute of Chinese Materia Medica, Chinese Academy of Chinese Medical Sciences, Beijing, 100700 China; 2 Guangdong Provincial Key Laboratory of New Drug Screening, School of Pharmaceutical Sciences, Southern Medical University, Guangzhou, 510515 China; 3 Laboratory Medicine, the First Affiliated Hospital of Gannan Medical University, Ganzhou, 341000 Jiangxi China; 4 Department of Geriatric Medicine, Shenzhen People’s Hospital, the Second Clinical Medical College, Jinan University and the First Affiliated Hospital, Southern University of Science and Technology, Shenzhen, 518020 Guangdong China; 5 Guangdong Provincial Key Laboratory of Proteomics, School of Basic Medical Sciences, Southern Medical University, Guangzhou, 510515 China; 6 Center for Reproductive Medicine, Dongguan Maternal and Child Health Care Hospital, Southern Medical University, Dongguan, 523125 Guangdong China; 7 Central People’s Hospital of Zhanjiang, Zhanjiang, 524037 Guangdong China

**Keywords:** Celastrol, Sepsis, Pyruvate kinase M2, High mobility group box 1, Aerobic glycolysis

## Abstract

**Background:**

Sepsis involves life-threatening organ dysfunction and is caused by a dysregulated host response to infection. No specific therapies against sepsis have been reported. Celastrol (Cel) is a natural anti-inflammatory compound that shows potential against systemic inflammatory diseases. This study aimed to investigate the pharmacological activity and molecular mechanism of Cel in models of endotoxemia and sepsis.

**Methods:**

We evaluated the anti-inflammatory efficacy of Cel against endotoxemia and sepsis in mice and macrophage cultures treated with lipopolysaccharide (LPS). We screened for potential protein targets of Cel using activity-based protein profiling (ABPP). Potential targets were validated using biophysical methods such as cellular thermal shift assays (CETSA) and surface plasmon resonance (SPR). Residues involved in Cel binding to target proteins were identified through point mutagenesis, and the functional effects of such binding were explored through gene knockdown.

**Results:**

Cel protected mice from lethal endotoxemia and improved their survival with sepsis, and it significantly decreased the levels of pro-inflammatory cytokines in mice and macrophages treated with LPS (*P* < 0.05). Cel bound to Cys424 of pyruvate kinase M2 (PKM2), inhibiting the enzyme and thereby suppressing aerobic glycolysis (Warburg effect). Cel also bound to Cys106 in high mobility group box 1 (HMGB1) protein, reducing the secretion of inflammatory cytokine interleukin (IL)-1β. Cel bound to the Cys residues in lactate dehydrogenase A (LDHA).

**Conclusion:**

Cel inhibits inflammation and the Warburg effect in sepsis via targeting PKM2 and HMGB1 protein.

**Supplementary Information:**

The online version contains supplementary material available at 10.1186/s40779-022-00381-4.

## Background

Sepsis, a major cause of morbidity and mortality, involves an excessive systemic inflammatory response to infection or injury, triggering multi-organ dysfunction [1, 2]. In sepsis, macrophages produce pro-inflammatory cytokines such as interleukin (IL)-1β, initiating an innate immune response to pathogens [3, 4]. These macrophages require additional energy to become activated, so they shift their glucose metabolism from oxidative phosphorylation to aerobic glycolysis, a shift known as the “Warburg effect”, which was first observed in proliferative cancer cells [5, 6]. The metabolites arising from such aerobic glycolysis may further regulate immune responses [7].

Several enzymes support aerobic glycolysis and may therefore be important in driving macrophage activity in sepsis. Pyruvate kinase M2 (PKM2) is one of the four isoforms of pyruvate kinase, which catalyzes the conversion of phosphoenolpyruvate to pyruvate [8, 9]. Lactate dehydrogenase A (LDHA) converts pyruvate to lactate [10]. PKM2-dependent aerobic glycolysis promotes IL-1β release in macrophages activated by lipopolysaccharide (LPS) [11, 12].

PKM2 also promotes the secretion of the high mobility group box 1 (HMGB1) protein [13], which mediates immune responses in activated macrophages and has already been identified as a potential therapeutic target in sepsis [14]. With three Cys residues that can be reduced to thiols [15, 16], HMGB1 must be in the appropriate redox state to bind to Toll-like receptor (TLR) 4 [17, 18], which participates in sepsis-induced multi-organ failure [19]. Secreted HMGB1 can bind to TLRs and the receptor of advanced glycation end products in order to stimulate the release of pro-inflammatory cytokines [20–22].

These considerations suggest the potential of mitigating inflammatory injury in sepsis and endotoxemia by inhibiting HMGB1 and enzymes involved in aerobic glycolysis [9, 10, 23]. Celastrol (Cel), a bioactive natural product from the plant *Tripterygium wilfordii* (Additional file 1: Fig. S1a) [24], has shown potential as an anti-inflammatory and immunosuppressive agent against systemic inflammation, rheumatoid arthritis, and metabolic diseases [25, 26], as well as against circulatory failure and acute hepatic dysfunction in sepsis [27–29]. On the other hand, at least one study has reported that Cel can actually aggravate inflammation and organ injury after LPS treatment [30]. Therefore, further work is needed to establish whether and how Cel may mitigate tissue injury due to endotoxemia or sepsis. Since Cel is known to target proteins by binding to thiol groups of Cys residues [31], we hypothesized that it may use this mechanism to work against endotoxemia and sepsis. This study explored this hypothesis in animal and cell culture models of these two conditions.

## Methods

### Reagents

Cel was obtained from Beijing Bethealth People Biomedical Technology (Beijing, China). Shikonin (SKN) was purchased from Selleck (Shanghai, China). LPS from *Escherichia coli* (*E. coli*) and prednisone (PNS) were obtained from Sigma (Co. St. Louis, USA). Antibodies against PKM2, LDHA, and IL-1β were obtained from Abcam (Cambridge, UK), as were recombinant human HMGB1, PKM1 and LDHA. Antibody against HMGB1 was obtained from CST (Boston, USA), antibody against PKM1 was obtained from Proteintech Group (Chicago, USA), and antibody against β-actin was obtained from Affinity Biosciences (Beijing, China). Enzyme-linked immunosorbent assay (ELISA) against mouse tumor necrosis factor-α (TNF-α), IL-1β, and IL-6 were purchased from Thermo Fisher (MA, USA). Assay kits for lactate dehydrogenase (LDH), aspartate aminotransferase (AST), alanine aminotransferase (ALT), uric acid (UA) and urea were obtained from Beijian Xinchuangyuan Technology (Beijing, China). Assay kits for mitochondrial stress and glycolysis were obtained from Agilent (California, USA) and used with the Seahorse Extracellular Flux Analyzer (Agilent, California, USA) [32].

### Experimental models of endotoxemia and sepsis

Animal experiments were approved by the China Animal Care and Use Committee (Aup-210320-WJG-001-01) and were conducted in accordance with Regulations on the Care and Use of the Laboratory Animal Center of Shenzhen People’s Hospital. Fifty-six male BALB/c mice (7–8 weeks old) were purchased from Guangdong Vital River Laboratory Animal Technology (Guangzhou, China). The experimental scheme is shown in Additional file 1: Fig. S1b. To study sepsis induced by cecal ligation puncture (CLP), 24 mice were divided into 3 groups (*n* = 8 per group): control group, CLP model group and CLP-Cel group. The mice in control group did not undergo CLP or receive Cel, mice in CLP model group underwent CLP, and mice in CLP-Cel group underwent CLP, followed 1 h later by intraperitoneal Cel (1 mg/kg) once a day for 1 d. Mouse mortality was recorded during the first 48 h after CLP, then animals were euthanized.

To study LPS-induced endotoxemia, 32 mice were randomly assigned to four groups (*n* = 8 per group): control group, model group, Cel group and PNS group. The mice in control group received neither Cel nor LPS; mice in model group received LPS (15 mg/kg) intraperitoneally to induce endotoxemia [12]; mice in Cel group received Cel intraperitoneally (1 mg/kg) once daily for 7 d, after which the animals were given LPS; and mice in PNS group received PNS (5 mg/kg) once daily for 7 d, followed 1 h later by LPS. PNS served as a positive control for anti-septic effects [33, 34]. Cel was prepared in normal saline. From all four groups, blood and major organs (intestine, kidney, liver, and lung) were collected and analyzed. Serum samples were stored at −80 °C before analysis.

#### Cytokine assays

Commercial ELISA was used according to the manufacturer’s instructions to measure concentrations of TNF-α, IL-1β, and IL-6 in serum from mice.

#### Serum and blood analyses

Levels of AST, ALT, urea, UA, and LDH were assayed using commercial kits and an automatic biochemistry analyzer (TBA-120FR, Toshiba, Japan). Blood cell types were quantified using an automatic five-category blood analyzer.

#### Hematoxylin–eosin (HE) staining

Tissue samples from mice were fixed in 4% paraformaldehyde, embedded in paraffin, sectioned, and stained with hematoxylin–eosin (HE).

### Macrophage culture and viability assays

RAW264.7 cells were obtained from the Chinese National Cell Bank (Beijing, China) and cultured in Dulbecco’s modified Eagle medium containing 10% fetal bovine serum (FBS) at 37 °C in an atmosphere of 5% CO_2_ and 95% relative humidity. RAW264.7 cells were seeded into 96-well plates at a density of 6 × 10^3^ cells/well and incubated for 12 h, after which Cel or celastrol-probe (Cel-P, 0.1–4.0 μmol/L) was added and the cultures were incubated another 24 h. Cell viability was assessed using the CCK-8 kit (Dojindo, Japan) according to the manufacturer’s instructions.

### Assays of cytokine secretion, extracellular acidification, and oxygen consumption by macrophages in culture

RAW264.7 cells were treated in the following groups: DMSO group, model group, Cel group, and SKN group. DMSO group was treated only with DMSO vehicle; Model group was treated with LPS (100 ng/ml) for 3 h; Cel group was treated with LPS (100 ng/ml) for 3 h, then fresh medium was added containing Cel (1 μmol/L) for 24 h; SKN group was treated with LPS (100 ng/ml) for 3 h, then fresh medium was added containing SKN (1 μmol/L) for 24 h.

The culture medium was harvested and assayed for secreted TNF-α, IL-1β, and IL-6 using ELISA kits, according to the manufacturer’s instructions. The cells were also harvested for further experiments.

In macrophages cultured as described above, the extracellular acidification rate (ECAR) and oxygen consumption rate (OCR) were determined using the Seahorse Extracellular Flux Analyzer. Data were analyzed using Seahorse XFe96 software (Agilent, California, USA).

### Protein labeling in situ and in vitro

The proteome of RAW264.7 cells in culture was labeled in situ as described [35, 36], and the ABPP experimental scheme is shown in Additional file 1: Fig. S2. Briefly, macrophages were incubated with LPS (100 ng/ml) for 3 h, then treated with Cel-P or DMSO for 2 h, and total cellular proteins were extracted. To equal amounts of protein was added freshly prepared “clickable reaction cocktail” [100 μmol/L Tris (3-hydroxypropyltriazolylmethyl) amine (THPTA), 1 mmol/L vitamin C sodium (NaVc), 50 μmol/L TAMRA-azide, 1 mmol/L CuSO_4_], and the mixture was incubated for 2 h at room temperature. Then acetone prechilled to −20 °C was added, and the precipitated proteins were resolubilized in 50 μl 1 × loading buffer by sonication and heating for 12 min at 95 °C. The proteins were fractionated by 10% SDS-PAGE, and proteins labeled by Cel-P were visualized using Azure Sapphire (RGB-NIR, USA). Finally, the gel was stained with Coomassie Brilliant Stain (Abcam, Cambridge, UK).

For labeling of recombinant purified protein in vitro, proteins were treated with Cel-P in phosphate-buffered saline (PBS) for 60 min at room temperature, then mixed with the clickable cocktail and incubated another 2 h at room temperature. Proteins were separated by SDS-PAGE, followed by visualization with Azure Sapphire RGBNIR.

In competitive protein labeling experiments, cells or proteins were first incubated with competitors for 30 min, then treated with Cel-P for 60 min. Subsequently, click chemistry and electrophoresis were carried out as described above.

### ABPP-based identification of targets

RAW264.7 cells were activated with LPS for 3 h, incubated another 30 min with or without Cel, then treated with Cel-P or DMSO for 2 h. Total protein was extracted, estimated using a BCA kit, and subjected to click chemistry for 2 h at room temperature [37]. Proteins were precipitated using acetone pre-chilled to −20 °C, resolubilized in PBS containing 1.5% SDS, and incubated with streptavidin beads for 4 h at room temperature. Streptavidin beads were washed gently with 5 ml PBS containing 1% SDS (twice), 0.1% SDS (once), 6 mol/L urea (three times) and PBS (twice).

Proteins bound to the streptavidin beads were eluted and separated by SDS-PAGE. Bands were excised, minced, reduced with dithiothreitol (DTT) and alkylated using iodoacetamide (IAA), and digested with trypsin overnight at room temperature. The resulting peptides were desalted on a C^18^ column, labeled using TMT label reagents (Thermo Scientific, MA, USA), and identified by liquid chromatography – tandem mass spectrometry (LC–MS/MS, Orbitrap Fusion Lumos, Thermo Scientific, MA, USA).

Proteins bound to streptavidin beads were fractionated by SDS-PAGE as described above and analyzed by Western blotting.

### Fluorescence staining

RAW264.7 cells were seeded into sterile glass-bottom dishes and stimulated by LPS for 3 h. Then cells were incubated with or without Cel-P at concentrations of 0.25, 0.5 or 1.0 μmol/L. Cells were fixed with 4% paraformaldehyde and permeabilized. Cells were treated with click reaction cocktail for 2 h, incubated with Hoechst dye, then washed twice with PBS.

After the click reaction, cells were further incubated at 4 °C overnight with antibodies against PKM2 (1:200) or HMGB1 (1:200), then with secondary antibodies and finally with Hoechst dye. Images were captured under a TCS SP8 SR confocal microscope (Leica, Munich, Germany).

In other experiments, RAW264.7 cells were seeded into sterile glass-bottom dishes, stimulated with LPS for 3 h, then incubated another 4 h with or without Cel (1 μmol/L). Cells were fixed with 4% paraformaldehyde, permeabilized, blocked with BSA, incubated at 4 °C overnight with antibody against HMGB1 (1:200), then incubated with secondary antibody (1:200), and finally with Hoechst dye (1:1000) and dye to label the cell membrane (1:1000). Cells were washed twice with PBS, and images were captured under the TCS SP8 SR microscope.

### Western blotting

Total proteins in RAW264.7 cells were extracted, separated by SDS-PAGE and electro-transferred to polyvinylidene fluoride (PVDF) membranes. Membranes were incubated with antibodies against PKM2 (1:1000), LDHA (1:1000), IL-1β (1:1000), HMGB1 (1:1000), PKM1 (1:1000) and β-actin (1:5000), followed by the corresponding secondary antibodies (1:5000). Antibody binding was visualized using enzyme-linked chemiluminescence (Thermo Fisher, MA, USA) and analyzed using ImageJ software (version 1.8.0, National Institutes of Health, Bethesda, MD, USA).

### Cellular thermal shift assay

The binding of Cel to potential protein targets was validated using cellular thermal shift assays (CESTA) [38]. LPS-activated RAW264.7 cells were lysed on ice for 30 min, total protein was extracted, then equal amounts (2 mg/ml, 1 ml) were incubated with Cel (20 μmol/L) or DMSO at room temperature for 50–60 min. The mixtures were aliquoted into 10 PCR tubes, which were heated to 10 different temperatures for 3 min, then incubated at 4 °C for 3 min in a thermal cycler (Applied Biosystems, Thermo Scientific). Samples were centrifuged at 20,000×*g* for 10 min, mixed with 1 × loading buffer, heated at 95 °C for 10 min, and subjected to Western blotting.

### Surface plasmon resonance

Surface plasmon resonance was used as described [39] to explore binding of Cel to potential protein targets (PKM2, LDHA, and HMGB1). Single-cycle kinetic runs were performed on a Biacore S200. Two-fold serial dilutions of Cel from 0.39 to 1000 μmol/L were passed over the CM5 series S sensor chip surface at 30 μl/min for 120 s, followed by 240 s of dissociation flow. The flow temperature was 25 °C. Data were analyzed using Biacore software. All curve baselines were adjusted to zero and aligned with the injection start time. The reference sensorgram was subtracted from experimental sensorgrams. The 1:1 binding model (Langmuir) was used to assess binding kinetics, yielding the association rate constant (*k*_a_) and dissociation rate constant (*k*_d_). The binding affinity (*K*_D_) was evaluated based on the concentration dependence of the steady-state response.

### Protein purification

DNA sequences encoding human wild-type PKM2 (GenBank Accession NP_002645.3) and its Cys424Ser mutant were cloned into pET28a (Sangon, Shanghai, China). Sequences encoding human HMGB1 (GenBank Accession NP_002119.1) or the A box domain or B box domain were cloned into pET-24d (Sangon, Shanghai, China). The plasmids were transformed into *E. coli* BL21 and protein expression was induced for 12 h at 16 °C using 0.4 mmol/L isopropyl-d-1-thiogalactopyranoside. Bacterial pellets were collected and lysed in binding buffer (20 mmol/L Tris–HCl, 200 mmol/L NaCl, 1 mmol/L PMSF, pH 8.0). Recombinant proteins were purified on a Ni–NTA column, eluted with imidazole, and concentrated on spin-columns. Recombinant protein purity and integrity were visualized using Coomassie Brilliant Blue.

### Activity assays of recombinant human LDHA and PKM2

The activities of purified recombinant human LDHA and PKM2 were measured in vitro with or without Cel at 10, 20 and 40 μmol/L, following the manufacturer’s instructions (Abcam, Cambridge, UK).

### Activity assays of recombinant human HMGB1

RAW264.7 cells were seeded into 6-well plates, incubated for 24 h, then treated with recombinant human HMGB1 or B box protein as well as Cel (1 μmol/L) for 24 h. Cells were harvested and assayed for intracellular IL-1β by Western blotting. The levels of IL-1β served as a marker of the activity of HMGB1 and B box protein.

### UV–visible absorption

The absorption spectrum of Cel in the UV–visible range (300–600 nm) was determined for Cel at 0–200 μmol/L in PBS. In addition, spectra were recorded for Cel (100 μmol/L) incubated with the following recombinant human proteins: A box, B box, PKM1, wild-type PKM2, and PKM2-Cys424Ser.

### Molecular docking of Cel with potential protein targets

The structure of Cel was downloaded from PubChem, and the three-dimensional structures of PKM2 (PDB: 4B2D) and LDHA (PDB: 4ZVV) were obtained from the RCSB PDB. The 3D protein structure of HMGB1 was modeled using Phyre2. Discovery Studio Client was used to add water and hydrogen atoms to proteins. Cel was docked onto these structures using AutoDock Tools, AutoDock Vina and Pyrx-0.8. The docking results were analyzed and visualized using Pymol.

### RNA interference and transfection

Sequences of short interfering (si)RNA targeting PKM2 (Additional file 2: Table S1) were designed and synthesized by Sangon. The siRNA and vectors were transfected into RAW264.7 cells using Lipofectamine 2000 (Thermo Fisher, MA, USA) according to the manufacturer’s instruction. Cells treated with siRNA were analyzed in assays of ECAR, OCR and Western blotting.

### Statistical analysis

All data were reported as mean ± standard error of the mean (SEM) from at least 3 biological replicates. GraphPad Prism 8.0 was used for statistical analyses. Inter-group differences were assessed for significance using one-way ANOVA or two group differences were assessed for significance using Student’s *t* test, unless otherwise mentioned. Inter-group differences in mouse survival were assessed using the Kaplan–Meier method. Differences associated with *P* < 0.05 were defined as statistically significant.

## Results

### Cel protects mice from endotoxemia and from sepsis-related mortality

**Fig. 1:**
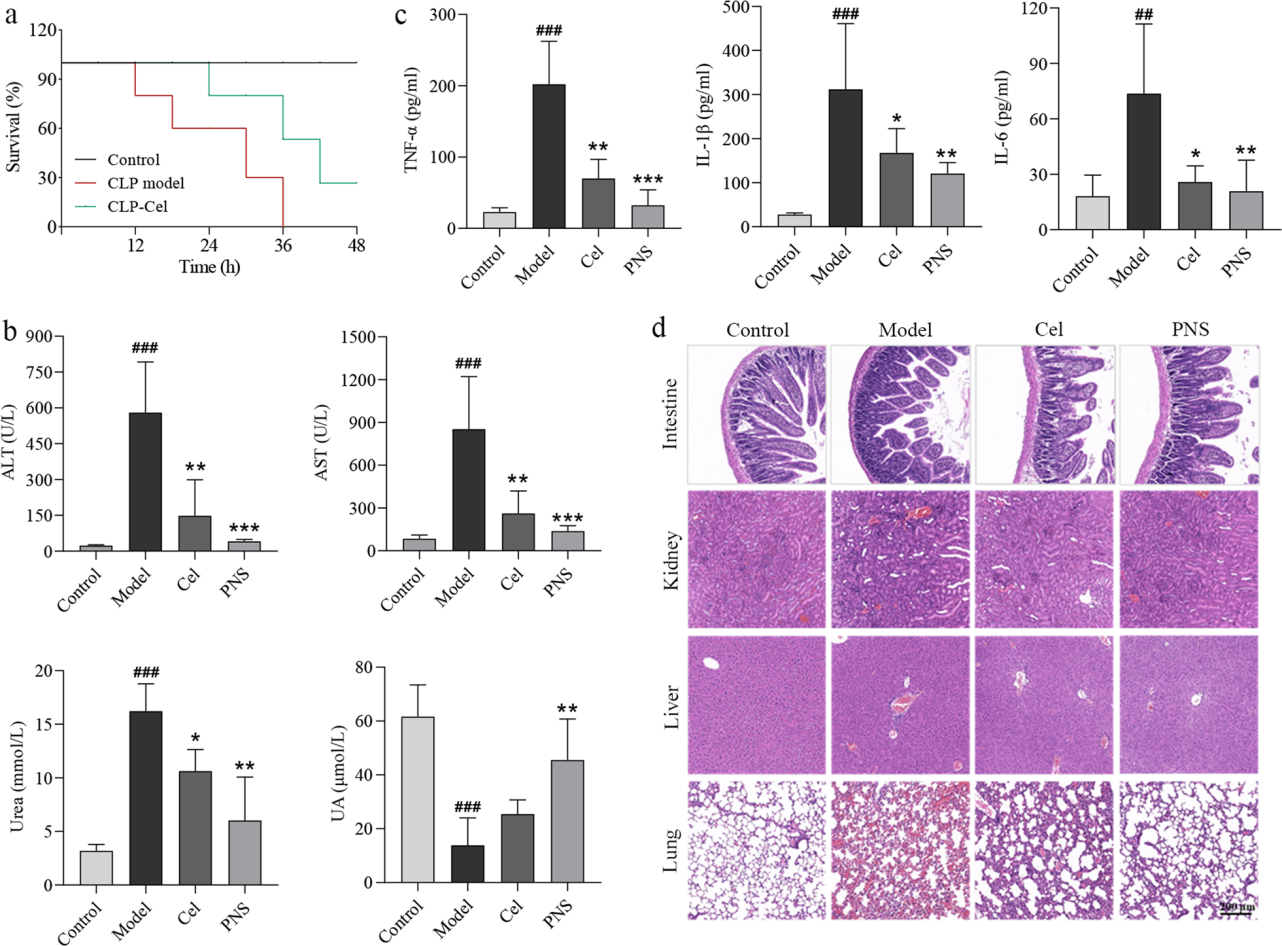
Celastrol (Cel) protects mice from experimental sepsis and endotoxic shock. **a** Survival rates of cecal ligation puncture (CLP) mice with or without Cel (1 mg/kg, i.p.). **b** Serum indicators of liver and kidney function in endotoxemic mice. **c** Release of the inflammatory cytokines TNF-α, IL-1β and IL-6 in endotoxemic mice. **d** Hematoxylin–eosin staining of intestine, kidney, liver and lung sections from endotoxemic mice. Scale bar = 200 μm. All data are expressed as mean ± SEM (*n* = 6). ^###^*P* < 0.001 vs. Control; *P < 0.05, **P < 0.01, ***P < 0.001 vs. Model. ALT alanine aminotransferase, PNS prednisone, AST aspartate aminotransferase, UA uric acid, TNF-α tumor necrosis factor-α, IL-1β interleukin-1β, IL-6 interleukin-6

We evaluated whether Cel could protect mice from sepsis and lethal endotoxemia. We used LPS to rapidly induce endotoxemia [40, 41]. As a complementary model, we used CLP to induce sepsis [40]. LPS induces early, transient release of pro-inflammatory factors [40, 41], whereas CLP induces a delayed, persistent release of such factors [42]. Because of these differences, we pretreated animals with celestrol before LPS administration, but we treated them with celestrol after CLP. During the first 48 h after CLP, compared to CLP model, Cel significantly improved the survival rates of mice in sepsis (Fig. 1a). In the endotoxemia model, pretreatment with Cel followed by LPS significantly reduced serum levels of ALT, AST and urea ((*P* < 0.01 or *P* < 0.05, Fig. 1b), while it increased the UA level, but the difference was not statistically significant (*P* > 0.05, Fig. 1b). Cel pretreatment also decreased the serum levels of TNF-α, IL-1β and IL-6 (*P* < 0.01 or *P* < 0.05, Fig. 1c). It significantly increased white blood cell count but reduced eosinophil count (*P* < 0.05 or *P* < 0.01, Additional file 1: Fig. S3a). Cel did not have obvious effects on numbers of red blood cells (*P* > 0.05, Additional file 1: Fig. S3a). HE staining of major tissues confirmed that Cel mitigated LPS-induced damage to intestine, kidney, liver and lung, similar to the protective effects of PNS (Fig. 1d, Additional file 1: Fig. S3b). Collectively, these data suggest that Cel protects mice from lethal sepsis and endotoxic shock, possibly by inhibiting the release of inflammatory cytokines.

### Cel inhibits proinflammatory cytokine release and the Warburg effect in macrophages

**Fig. 2:**
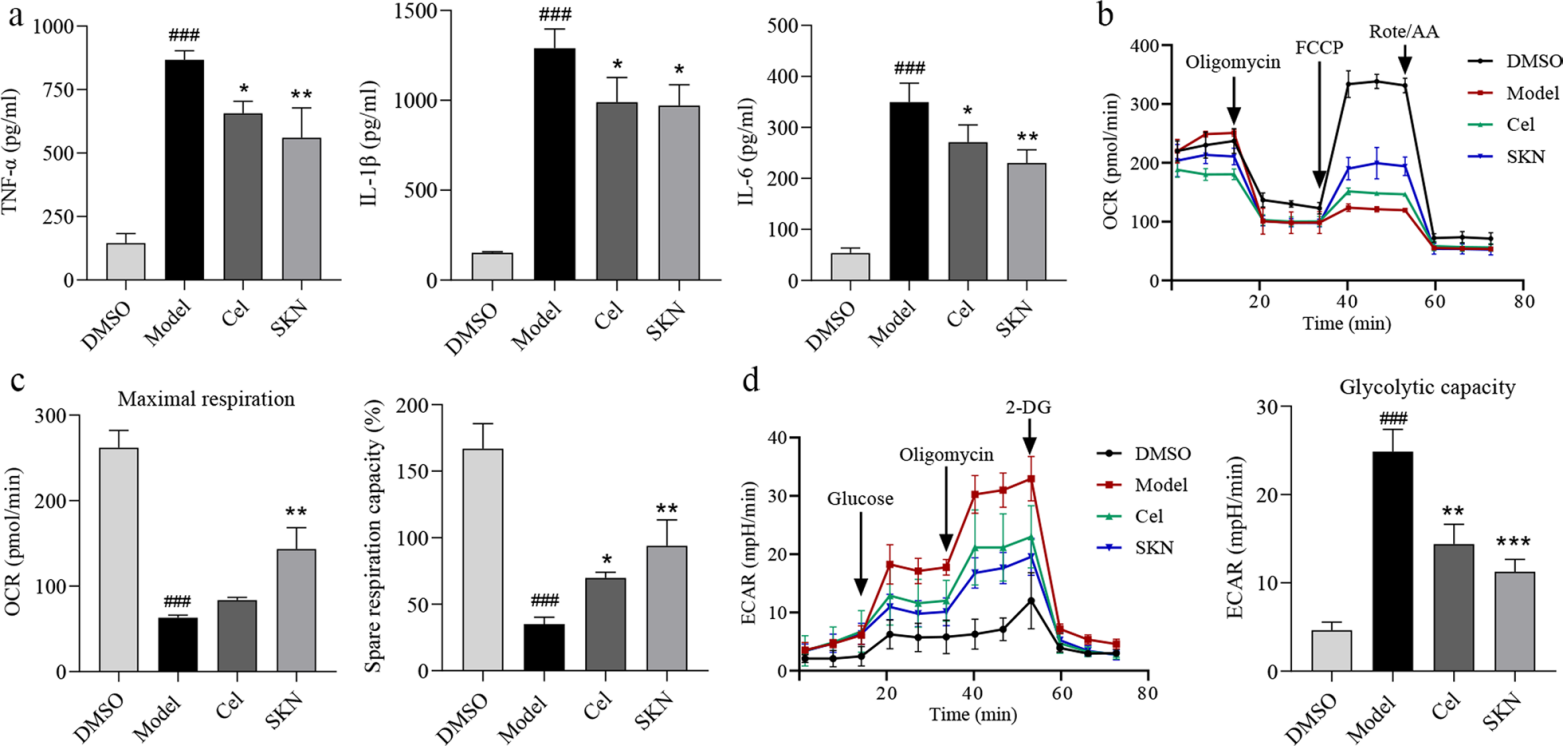
Celastrol (Cel) inhibits inflammatory response by suppressing the Warburg effect in LPS-activated macrophages. The grouping as follows: DMSO, Model (LPS 100 ng/ml), Cel (LPS 100 ng/ml + Cel 1 μmol/L), SKN (LPS 100 ng/ml + shikonin 1 μmol/L). **a** Release of the pro-inflammatory cytokines TNF-α, IL-1β and IL-6. **b** Oxygen consumption rate (OCR). **c** Maximal respiration and spare respiration capacity of OCR. **d** Extracellular acidification rate (ECAR). All data are expressed as mean ± SEM (*n* = 3). ^###^*P* < 0.001 vs. DMSO; *P < 0.05, **P < 0.01, ***P < 0.001 vs. Model. LPS lipopolysaccharide, SKN shikonin, TNF-α tumor necrosis factor-α, IL-1β interleukin-1β, IL-6 interleukin-6, FCCP p-trifluoromethoxy carbonyl cyanide phenylhydrazone, Rote/AA rotenone/antimycin A, 2-DG 2-deoxy-glucose

In sepsis, macrophages differentiate into an M1 lineage that produces inflammatory cytokines [11]. Cel has been shown to inhibit macrophage M1 polarization [43]. Consistently, we found that Cel suppressed the release of proinflammatory cytokines TNF-α, IL-1β, and IL-6 from LPS-activated macrophages in culture (*P* < 0.05, Fig. 2a). At the same time, we found that Cel reversed the LPS-induced decrease in OCR, similar to the effects of SKN (Fig. 2b–c, Additional file 1: Fig. S4a). Similarly, Cel suppressed ECAR including glycolytic capacity, like SKN (Fig. 2d, Additional file 1: Fig. S4b). Collectively, these results demonstrate that Cel dampens the release of proinflammatory cytokines and re-balances glycolytic metabolism in LPS-activated macrophages.

### Cel directly binds PKM2 and HMGB1

**Fig. 3:**
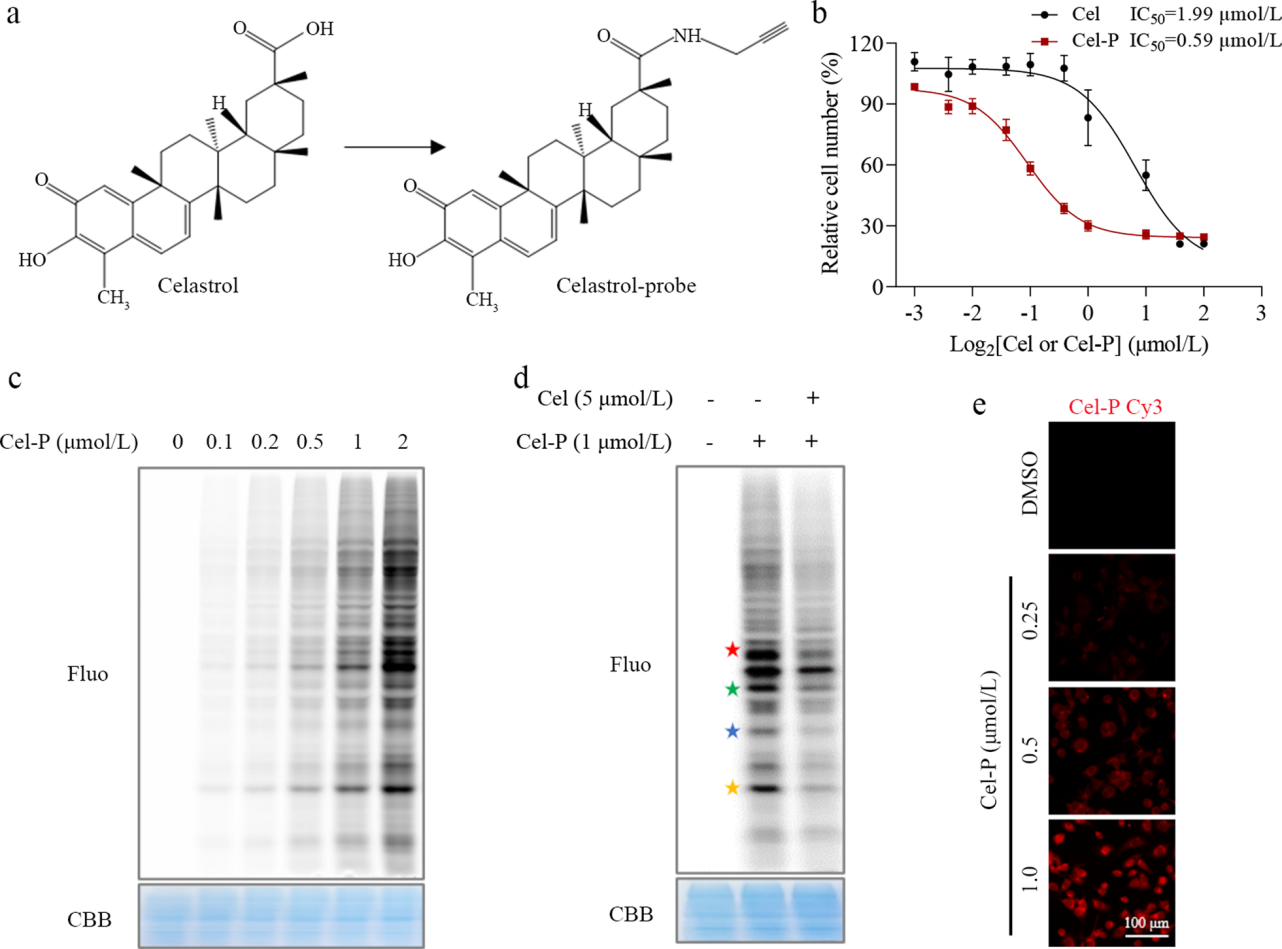
Identification of potential target proteins of celastrol (Cel) through ABPP combined with LC–MS/MS. **a** Chemical structure of Cel and celastrol-probe (Cel-P). **b** Dose-dependent inhibition of RAW264.7 cell proliferation by Cel and Cel-P. **c** Dose-dependent labeling of proteins by Cel-P in LPS-activated macrophages. **d** Competition between Cel and Cel-P for protein binding in situ (red star: 58 kD, green star: 43 kD, blue star: 37 kD, yellow star: 25 kD). **e** Imaging of intracellular Cel-P. Scale bar = 100 μm. ABPP activity-based protein profiling, LC–MS/MS liquid chromatography – tandem mass spectrometry, Fluo fluorescence, CBB coomassie brilliant blue

To understand how Cel exerts its anti-inflammatory effects, we identified its potential target proteins in LPS-activated macrophages. For these ABPP experiments, we synthesized a Cel-P with a clickable alkyne tag (Fig. 3a), and we verified that its inhibitory effects were similar to those of Cel (Fig. 3b). We incubated LPS-activated macrophages with Cel-P, and the proteins subsequently labeled by Cel-P were click-conjugated to the fluorescent dye TAMRA-azide (Fig. 3c). The specificity of Cel-P binding was confirmed by showing that free Cel competed effectively with Cel-P for fluorescent labeling (Fig. 3d). Using a fluorescently labeled Cel-P, we found that the probe was distributed throughout the cell (Fig. 3e), allowing a complete search of intracellular protein targets for celestrol.

**Fig. 4:**
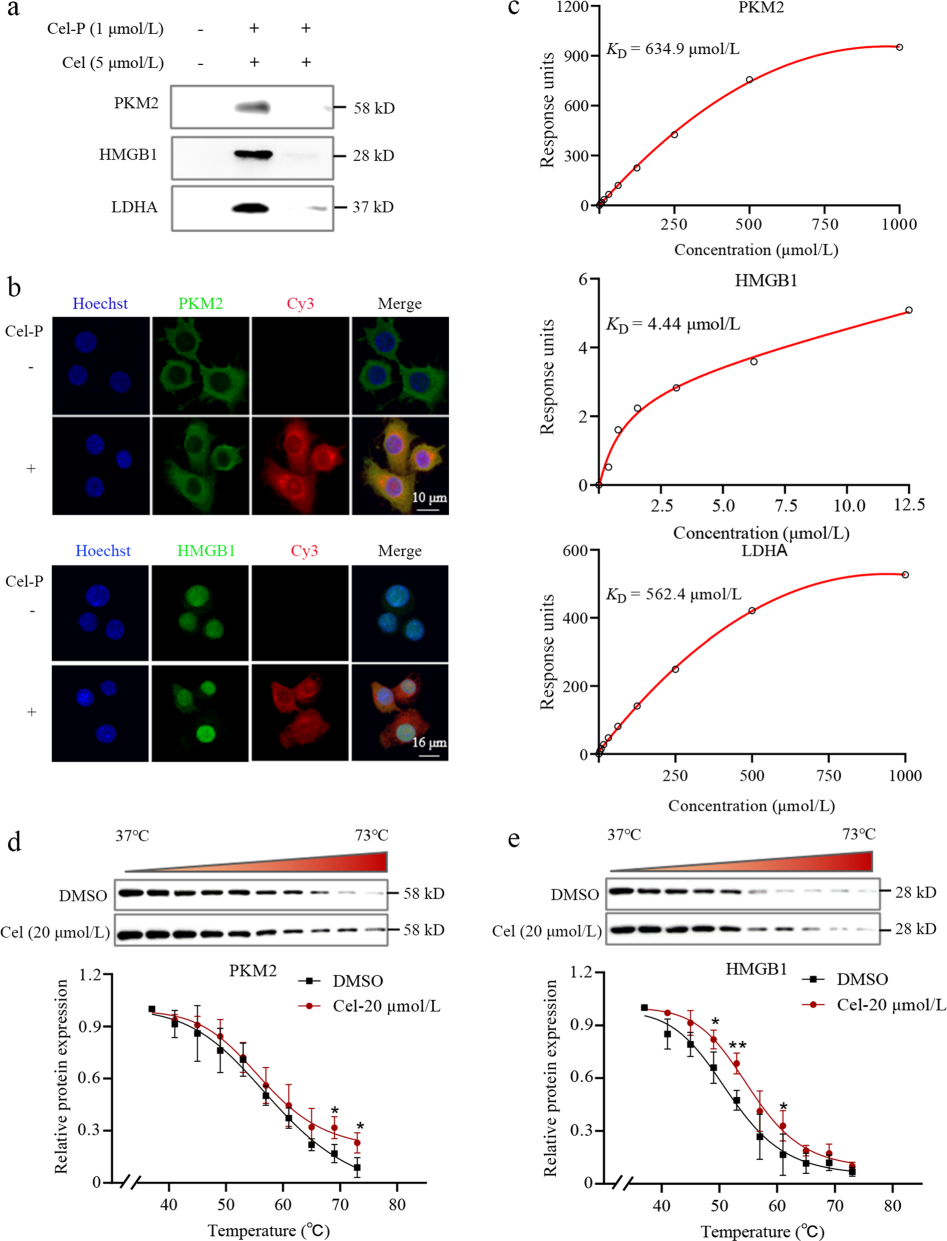
Celastrol (Cel) directly targets PKM2 and HMGB1. **a** Western blotting of pull-downs to verify Cel binding to PKM2, HMGB1 and LDHA in situ. **b** Immunofluorescence staining of PKM2 and HMGB1 (green) and Cel-P click-conjugated to a TAMRA dye (red). Scale bar = 10 μm or 12 μm. **c** Surface plasmon resonance studies of the binding affinity of Cel with recombinant human PKM2, HMGB1 and LDHA. **d** Cellular thermal shift assays to verify Cel binding to PKM2. **e** Cellular thermal shift assays to verify Cel binding to HMGB1 (*n* = 3). *P < 0.05, **P < 0.01 vs. 37 °C. Cel-P celastrol-probe, PKM2 pyruvate kinase M2, HMGB1 high mobility group box 1, LDHA lactate dehydrogenase A

Proteins that bound to Cel-P were click-reacted with biotin-azide, enriched on avidin-agarose beads, separated on SDS-PAGE, and identified by LC–MS/MS (Table 1). We confirmed in pull-down experiments that Cel-P bound PKM2, HMGB1 and LDHA, and this binding was outcompeted by excess Cel (Fig. 4a). Also consistent with such binding, PKM2 and HMGB1 co-localized with Cel-P (Fig. 4b). Surface plasmon resonance indicated that Cel bound to the three recombinant human proteins with *K*_D_ values of 634.9 μmol/L for PKM2, 4.44 μmol/L for HMGB1, and 562.4 μmol/L for LDHA (Fig. 4c). As further confirmation of binding, we showed in CETSA that Cel heat-stabilized PKM2 and HMGB1 (Fig. 4d-e). These data strongly suggest that in LPS-activated macrophages, Cel binds to PKM2 and HMGB1, and that LDHA may also be a target of Cel.

**Table 1:** Characteristics of key proteins in this study

Gene	Sum score	PSMs	Peptides	Cov (%)	MW (kD)
*PKM2*	166.2	52.0	21.0	49	57.8
*ACTB*	105.2	47	16.0	51	41.7
*LDHA*	41.0	15.0	7.0	26	36.5
*HMGB1*	24.5	11.0	4.0	20	24.9

*PSM* peptide-spectrum matches; *Cov* Coverage; *MW* molecular weight

### Cel inhibits pyruvate kinase activity and the proinflammatory activity of HMGB1

**Fig. 5:**
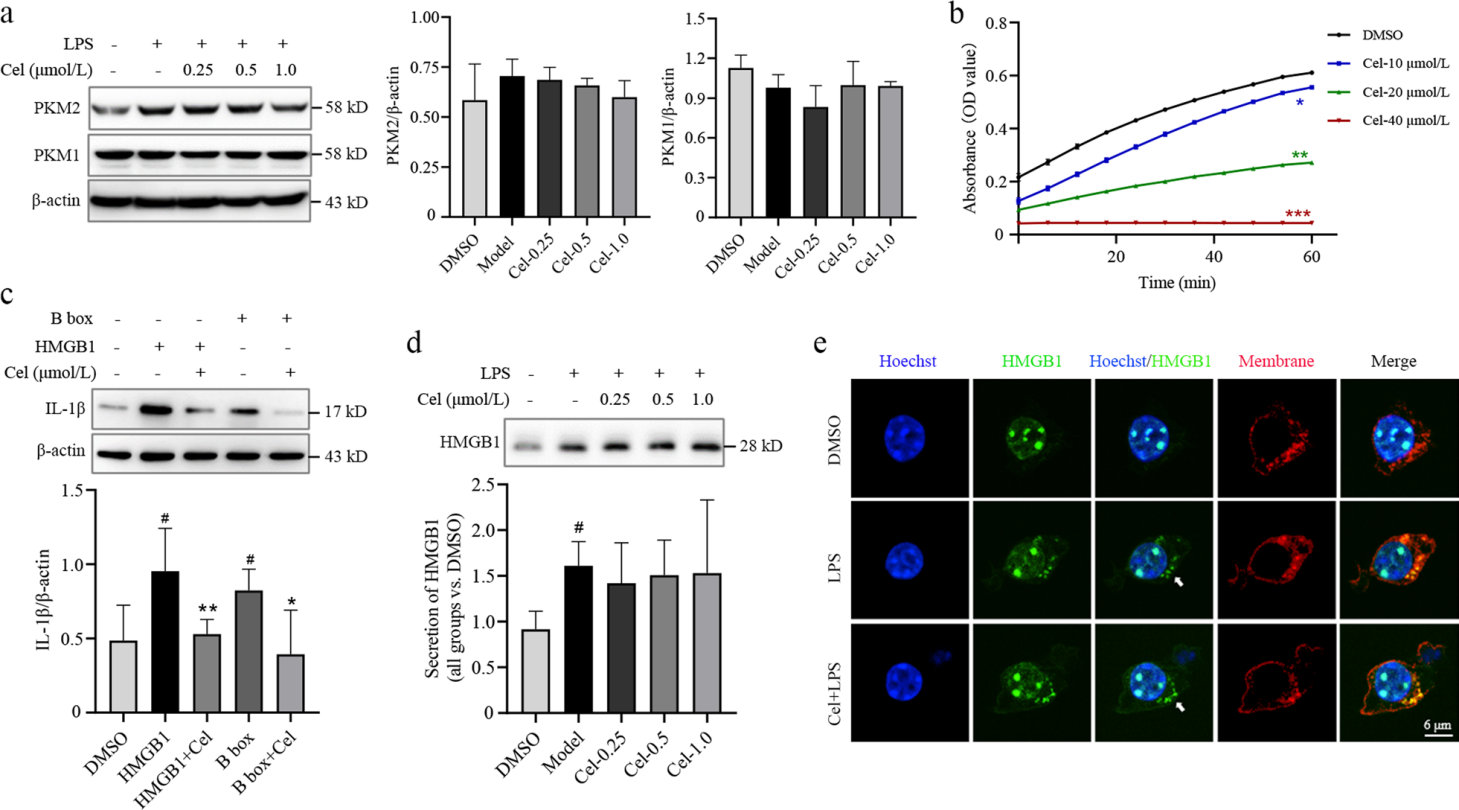
Celastrol (Cel) suppresses the enzymatic activity of pyruvate kinase and the proinflammatory activity of HMGB1. **a** Western blotting against PKM1 and PKM2 in macrophages treated or not with Cel at 0.25, 0.5 or 1.0 μmol/L. The corresponding densitometry is shown (*n* = 3). **b** Catalytic activity of pyruvate kinase with or without Cel treatment at 10, 20 and 40 μmol/L, *n* = 3, *P < 0.05, **P < 0.01, ***P < 0.001 vs. DMSO. **c** The contribution of HMGB1 to IL-1β expression, as determined by Western blotting. Assays were set up with HMGB1 at 0.8 μg/ml; B box, 0.8 μg/ml; and Cel, 0.2 μmol/L (*n* = 3). ^#^*P* < 0.05 vs. DMSO; *P < 0.05, **P < 0.01 vs. HMGB1 or B box. **d** Secretion of HMGB1 from macrophages treated or not with Cel at 0.25, 0.5 or 1.0 μmol/L, as determined using Western blotting (*n* = 3). ^#^*P* < 0.05 vs. DMSO. **e** Immunofluorescence staining with antibody against HMGB1 (green) or a dye targeting the cell membrane (red). Scale bar = 6 μm. LPS lipopolysaccharide, PKM2 pyruvate kinase M2, PKM1 pyruvate kinase M1, HMGB1 high mobility group box 1, IL-1β interleukin-1β

In LPS-activated macrophages, Cel did not significantly affect expression of PKM1 but it did slightly reverse the LPS-induced upregulation of PKM2, although this change did not achieve significance (*P* > 0.05, Fig. 5a, Additional file 1: Fig. S5). Nevertheless, we further explored whether Cel affects PKM2, and we found that it inhibited catalytic activity in a dose-dependent manner (*P* < 0.05, Fig. 5b).

HMGB1 contains a DNA-binding domain (A box), binding domain (B box) and a C-terminal. The redox state of these Cys-containing domains determines whether HMGB1 can bind target promoters or TLR4 [17], and since Cel can react with Cys residues, we examined whether Cel can prevent HMGB1 from activating secretion of cytokines. Indeed, incubating full-length HMGB1 or its B box with Cel significantly downregulated IL-1β (*P* < 0.05 or *P* < 0.01, Fig. 5c), Cel did not affect the secretion of HMGB1 (*P* > 0.05, Fig. 5d). Nevertheless, Cel did reverse the ability of LPS to shuttle HMGB1 out of the nucleus (Fig. 5e). These results suggest that Cel weakens the ability of HMGB1 to induce cytokine secretion, while also inhibiting pyruvate kinase. In this way, Cel simultaneously antagonizes the inflammation and Warburg effect that drive tissue injury in sepsis.

### Cel binds to Cys in PKM2 and HMGB1

**Fig. 6:**
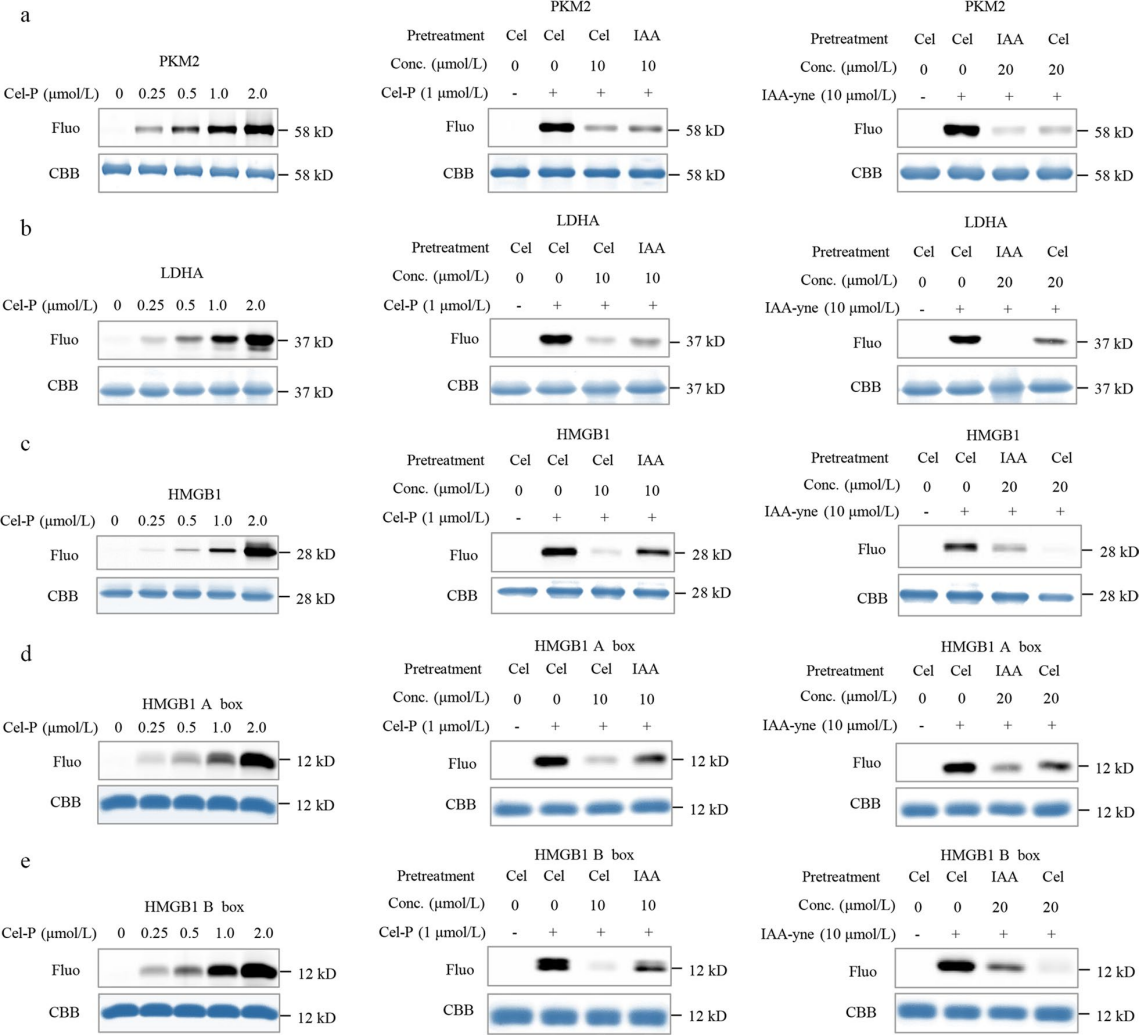
Celastrol (Cel) binds to Cys residues in PKM2, LDHA and HMGB1. **a** Recombinant human PKM2 labeled by celastrol-probe (Cel-P) or alkyne-tagged IAA in the presence or absence of the competitors Cel or IAA. **b** Recombinant human LDHA labeled by celastrol-probe (Cel-P) or alkyne-tagged IAA in the presence or absence of the competitors Cel or IAA. **c** Recombinant human HMGB1 labeled by celastrol-probe (Cel-P) or alkyne-tagged IAA in the presence or absence of the competitors Cel or IAA. **d** Recombinant human HMGB1 A box labeled by celastrol-probe (Cel-P) or alkyne-tagged IAA in the presence or absence of the competitors Cel or IAA. **e** Recombinant human HMGB1 B box labeled by celastrol-probe (Cel-P) or alkyne-tagged IAA in the presence or absence of the competitors Cel or IAA. PKM2 pyruvate kinase M2, LDHA lactate dehydrogenase A, HMGB1 high mobility group box 1, Conc. concentration, Fluo fluorescence, CBB coomassie brilliant blue, IAA iodoacetamide

Recombinant human proteins PKM2, LDHA and PKM1 were incubated with Cel-P, followed by click reaction with a fluorescent dye. These proteins were labeled with Cel-P in a dose-dependent manner (Fig. 6a–b, Additional file 1: Fig. S6a). Next, the three recombinant proteins were incubated with excess competing Cel or the Cys-alkylator IAA, then treated with Cel-P, and finally conjugated to a fluorescence dye. As expected, Cel and IAA competed with Cel-P for binding to PKM2 and LDHA (Fig. 6a–b), and they similarly prevented IAA that had been modified with a clickable alkyne tag to react with these two proteins (Fig. 6a–b). Collectively, these results suggest that Cel binds to Cys residues in PKM2 and LDHA.

Cel-P labeled recombinant human HMGB1 (Fig. 6c), the A box, and the B box in a dose-dependent manner (Fig. 6d–e). Cel or IAA competed with Cel-P or the alkyne-tagged IAA for labeling full-length HMGB1 as well as the A box and B box domains (Fig. 6c and e). These results are consistent with the fact that HMGB1 contains three Cys residues: Cys23, Cys45 in the A box domain, and Cys106 in the B box domain. Overall, our data suggest that Cel binds to Cys residues in HMGB1.

**Fig. 7:**
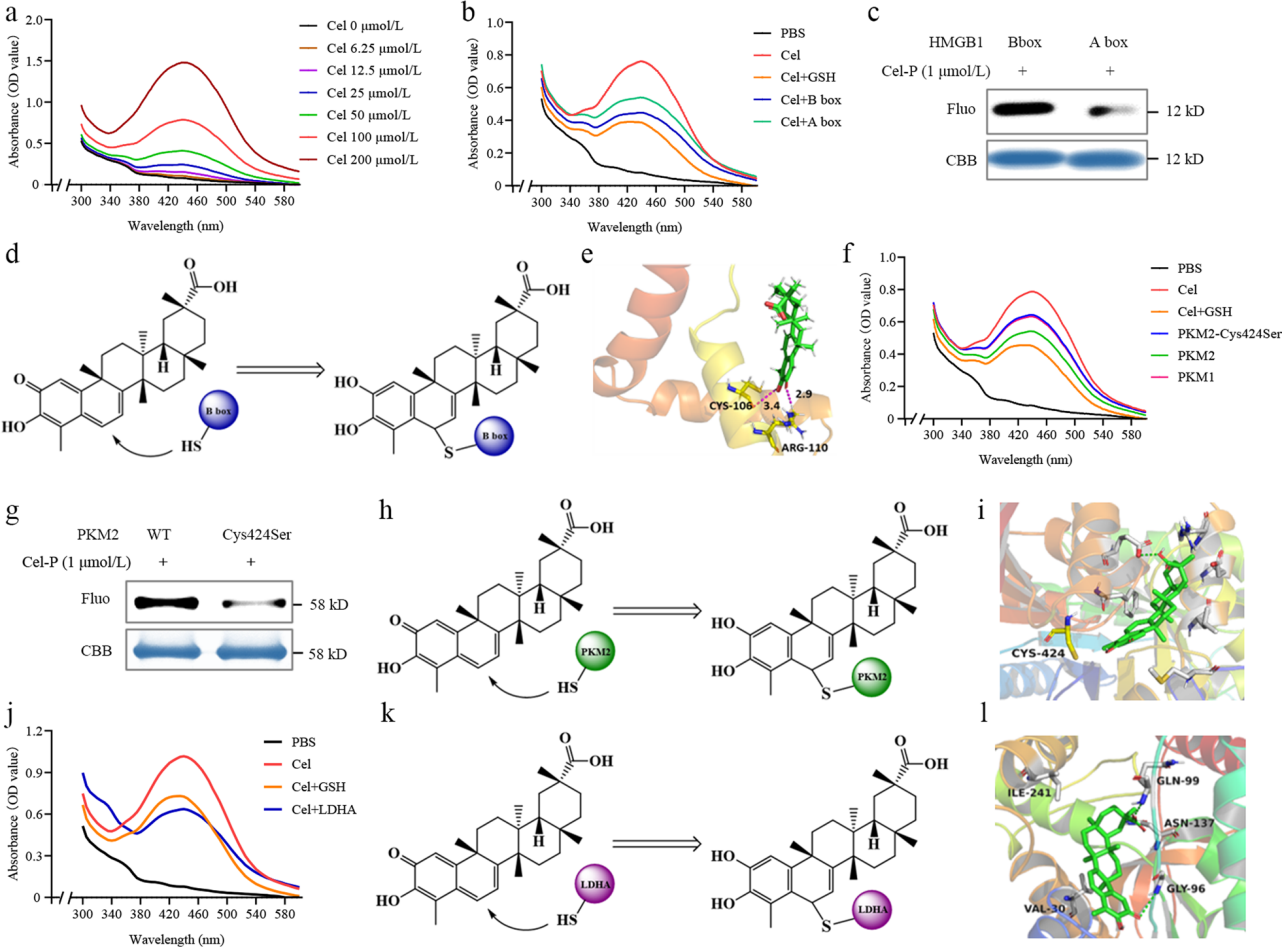
Celastrol (Cel) reacts with Cys residues in PKM2 and HMGB1 to inhibit their activities. **a** Absorption spectra of Cel at 6.25–200 μmol/L. **b** Absorption spectra of 100 μmol/L Cel in the presence or absence of GSH, HMGB1 A box, or B box. **c** Fluorescence intensity of recombinant A box and B box incubated with celastrol-probe (Cel-P) and then click-reacted with a fluorescent dye. **d** Scheme depicting Cel binding to B box. **e** Molecular docking model of Cel binding to HMGB1. **f** Absorption spectra of 100 μmol/L Cel in the presence or absence of GSH, PKM1, wild-type PKM2, or PKM2-Cys424Ser. **g** Fluorescence intensity of recombinant wild-type PKM2 and PKM2-Cys424Ser incubated with Cel-P and then click-reacted with a fluorescent dye. **h** Scheme depicting Cel binding to PKM2. **i** Molecular docking model of Cel binding to PKM2. **j** Absorption spectra of 100 μmol/L Cel in the presence or absence of GSH and LDHA. **k** Scheme of Cel binding to LDHA. **l** Molecular ducking model of Cel binding to LDHA. PBS phosphate-buffered saline, GSH glutathione, LDHA lactate dehydrogenase A, PKM2 pyruvate kinase M2, HMGB1 high mobility group box 1, Fluo fluorescence, CBB coomassie brilliant blue

As a complementary approach to evaluate the binding of Cel to potential targets, we took advantage of the fact that it absorbs strongly at 430–450 nm (Fig. 7a) and that this absorption decreases when Cel binds to, and reacts with, the thiol groups in glutathione [31]. Similarly, we found that UV–visible absorption by Cel decreased upon mixing with the B box domain, and to a lesser extent upon mixing with the A box domain (Fig. 7b). Under the same conditions, Cel-P labeled the B box to a larger extent than it labeled the A box (Fig. 7c). Given that atom C-6 in Cel undergoes Michael addition [31], we hypothesized that C-6 of Cel covalently binds to Cys106 in HMGB1, thereby inhibiting its proinflammatory activity (Fig. 7d). Molecular docking indicated the possibility that Cel binds to Cys106 in the B box domain of HMGB1 (Fig. 7e).

Next, we examined potential binding sites of Cel in PKM2. Mutating Cys424 to Ser in PKM2 increased its UV–visible absorption (Fig. 7f) and decreased Cel-P labeling (Fig. 7g–h), suggesting that Cel binds covalently to Cys424. Molecular docking suggested the possibility that Cel can bind to PKM2 in its substrate binding pocket (Fig. 7i).

We examined potential binding of Cel to LDHA. Mixing the two proteins reduced UV absorption by Cel, analogously to what happens when Cel is mixed with glutathione (Fig. 7j–k). In addition, Cel inhibited the activity of recombinant human LDHA (Additional file 1: Fig. S6a), and it reduced serum levels of LDH in mice treated with Cel (*P* < 0.01, Additional file 1: Fig. S6b). Molecular docking also suggested the possibility that Cel can bind to LDHA in its substrate binding pocket (Fig. 7l). These findings strongly suggest that Cel directly targets specific cysteines in HMGB1, PKM2, and LDHA in LPS-activated macrophages.

### Cel inhibits PKM2 signaling to attenuate LPS-induced inflammatory responses and glycolysis

**Fig. 8:**
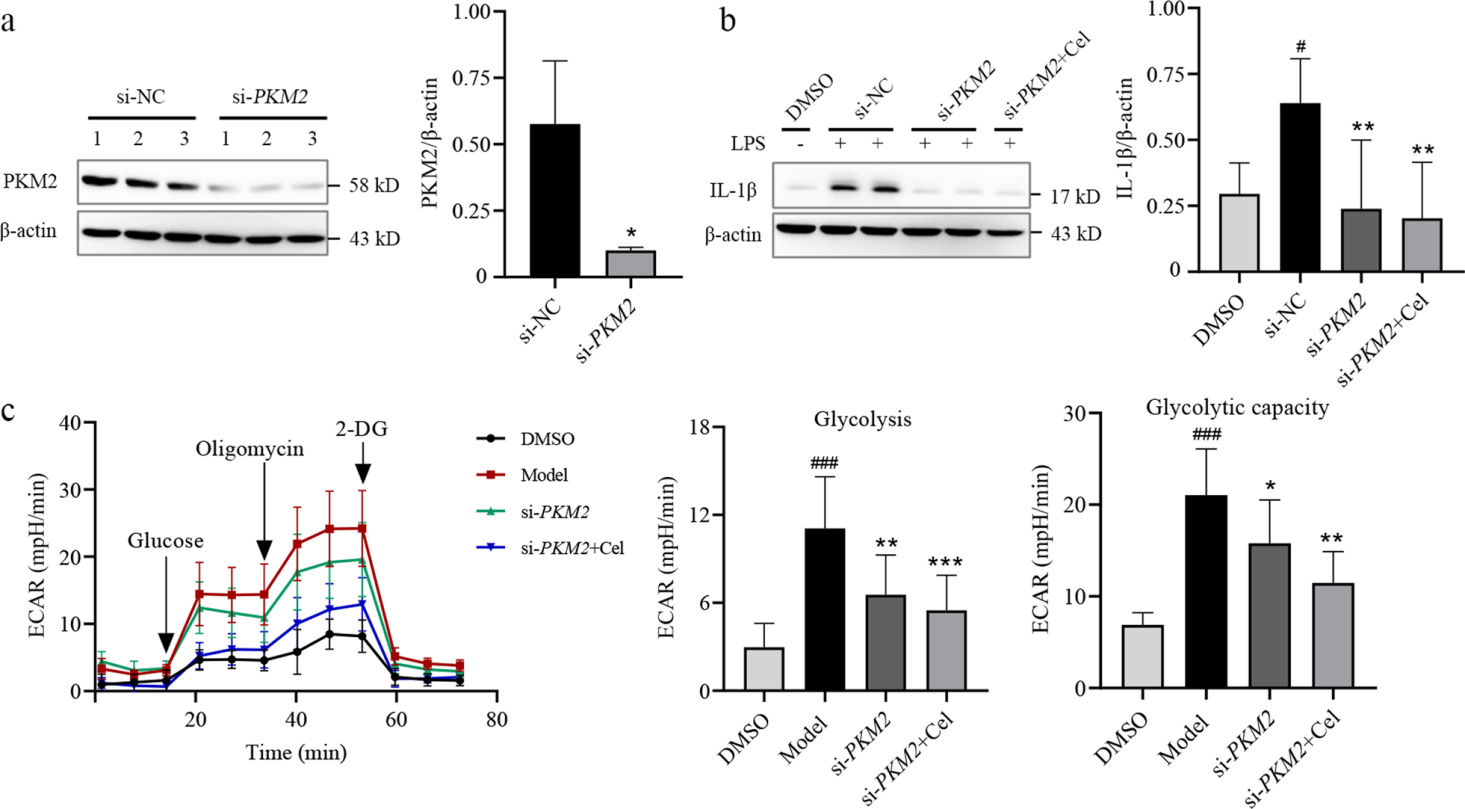
Celastrol (Cel) attenuates lipopolysaccharide (LPS)-induced inflammatory response and glycolysis. **a** Transfection of macrophages with short interfering (si)RNA against PKM2, and Western blotting with densitometry (*n* = 3). *P < 0.05 vs. si-NC. **b** IL-1β levels in cells transfected with si-*PKM2* or si-NC and cultured in the presence or absence of Cel at 0.5 μmol/L (*n* = 3). ^#^*P* < 0.05 vs. DMSO; **P < 0.01 vs. si-NC. **c** ECAR of cells transfected with si-*PKM2* in the presence or absence of Cel at 0.5 μmol/L (*n* = 3). ^###^*P* < 0.001 vs. DMSO; *P < 0.05, **P < 0.01, ***P < 0.001 vs. Model. PKM2 pyruvate kinase M2, IL-1β interleukin-1β, ECAR extracellular acidification rate, 2-DG 2-deoxy-glucose, NC negative control

To explore whether Cel inhibits glycolysis by targeting PKM2-dependent signaling, we used siRNA to knock down *PKM2* in LPS-activated macrophages (Fig. 8a). This knockdown significantly downregulated IL-1β expression (*P* < 0.01, Fig. 8b), and partially reversed LPS-induced inflammatory responses in the macrophage cultures. Furthermore, *PKM2* knockdown partially reversed the LPS-induced Warburg effect, which Cel further reversed (Fig. 8c). Collectively, our findings show that Cel acts via PKM2 signaling to attenuate proinflammatory responses and aerobic glycolysis in LPS-activated macrophages.

## Discussion

**Fig. 9:**
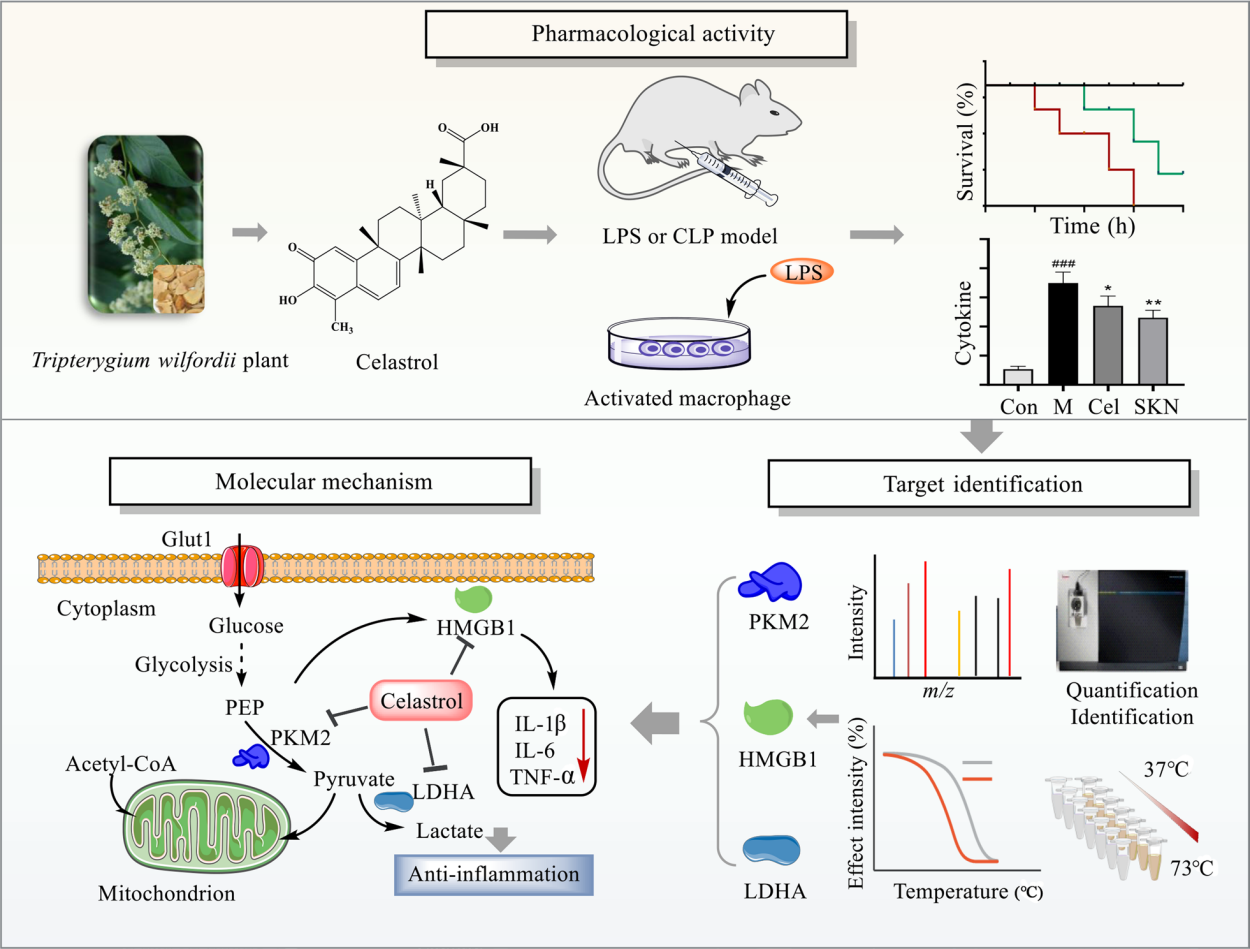
Summary of the key findings in this paper. Celastrol (Cel) protects mice from experimental sepsis and endotoxic shock by inhibiting secretion of pro-inflammatory cytokines. Cel directly targets PKM2, HMGB1, and LDHA, as shown using activity-based protein profiling and cellular thermal shift assays. Cel dampens the inflammatory response and glycolysis in LPS-activated macrophages by repressing the activities of PKM2 and HMGB1. LPS lipopolysaccharide, CLP cecal ligation puncture, PEP phosphor-enol-pyruvate, PKM2 pyruvate kinase M2, HMGB1 high mobility group box 1, LDHA lactate dehydrogenase A, IL-1β interleukin-1β, IL-6 interleukin-6, TNF-α tumor necrosis factor-α

Although our understanding of sepsis has greatly improved, effective treatments or preventive strategies are still urgently needed [44]. Here we show in a mouse model that the natural product Cel attenuates production of pro-inflammatory cytokines that drive tissue damage in sepsis, leading to better parameters of liver and kidney function as well as overall survival. Our experiments in cell culture and in vivo identified several proteins whose Cys residues are targeted by Cel, and through which the compound inhibits damaging inflammatory responses and the Warburg effect that helps sustain them (Fig. 9).

PKM2 is a crucial driver of the inflammatory response and Warburg effect in sepsis [11–13], and inhibiting the enzyme can suppress aerobic glycolysis and mitigate septic injury [45]. Here we found that Cel remarkably reduced release of the pro-inflammatory cytokines TNF-α, IL-1β, and IL-6 from LPS-activated macrophages, in part by inhibiting PKM2. Consistent with these observations, aerobic glycolysis is known to activate the inflammasome and the release of HMGB1, TNF-α, IL-1β, and IL-6 [12]. Therefore, Cel may attenuate inflammatory responses in sepsis by targeting PKM2 or other enzymes, thereby inhibiting aerobic glycolysis. Consistent with this hypothesis, we found that knocking down *PKM2* downregulated IL-1β and aerobic glycolysis, consistent with previous studies [12, 13].

Building on our previous experiences using ABPP to identify cellular targets of natural products and small molecules [46–48], we applied the same technique here to show that Cel could bind directly to PKM2, LDHA, and HMGB1 in LPS-activated macrophages. We verified our findings using surface plasmon resonance and CETSA. Cel inhibited the activities of PKM2 and LDHA, key enzymes in glycolysis [6, 9, 49], in a dose-dependent manner. Furthermore, we found that Cel inhibits the ability of HMGB1 to induce production of pro-inflammatory cytokines. HMGB1 has already been identified as a therapeutic target in sepsis [14, 50], and several agents that improve survival in sepsis or endotoxemia work by inhibiting secretion of HMGB1 [23, 51–53]. In contrast, Cel did not reduce HMGB1 secretion from LPS-activated macrophages, suggesting that the compound inhibits HMGB1-driven injury via a different mechanism.

Using a variety of labeling and competition approaches, we provide evidence that the reactive atom C-6 of Cel binds to Cys424 of PKM2 via Michael addition, and that Cel binds to Cys106 of HMGB1, whose redox state is known to be important for TLR4 activation and induction of cytokine release [17]. These findings provide testable hypotheses for exploring Cel’s mechanism of action and perhaps for improving its potency.

## Conclusions

We explored the pharmacological activity and protein targets of Cel in animal and cell-culture models of endotoxemia and sepsis. Cel can protect mice from lethal endotoxemia and sepsis, and it appears to do so by targeting glycolytic PKM2 in order to inhibit aerobic glycolysis and release of pro-inflammatory cytokines. Cel may bind to Cys106 in HMGB1 and prevent it from inducing the production of cytokines. Cel also appears to inhibit the activity of LDHA. Altogether, the data suggest that Cel mitigates sepsis-induced tissue injury by suppressing inflammatory responses and the Warburg effect that helps support them. Future studies should continue to explore the mechanism of action of Cel as well as optimize the dosing concentrations and timing in order to move the compound closer to the clinic.

## Supplementary Information

**Additional file 1: Fig. S1.** Chemical structure of celastrol (Cel) and scheme of animal experiments. **Fig. S2.** Overall workflow of activity-based protein profiling for identifying potential targets of celastrol (Cel). **Fig. S3.** Celastrol (Cel) protects mice from experimental sepsis and endotoxic shock. **Fig. S4.** Celastrol (Cel) inhibits the Warburg effect in LPS-induced macrophages. **Fig. S5.** Celastrol (Cel) binds to Cys residues of PKM1. **Fig. S6.** Celastrol (Cel) binds to Cys residues of LDHA and inhibits its activity.

**Additional file 2: Table S1.** Short interfering RNA sequences used to knockdown the *PKM2* gene in macrophages.

## Data Availability

Not applicable.

## Abbreviations

ABPP: Activity-based protein profiling
ALT: Alanine aminotransferase
AST: Aspartate aminotransferase
Cel-P: Celastrol-probe
CETSA: Cellular thermal shift assays
CLP: Cecal ligation puncture
DTT: Dithiothreitol
ECAR: Extracellular acidification rate
FBS: Fetal bovine serum
HE: Hematoxylin–eosin
HMGB1: High mobility group box 1
IAA: Iodoacetamide
IL-1β: Interleukin-1β
LC–MS/MS: Liquid chromatography–tandem mass spectrometry
LDHA: Lactate dehydrogenase A
LDH: Lactate dehydrogenase
LPS: Lipopolysaccharide
OCR: Oxygen consumption rate
PBS: Phosphate-buffered saline
PNS: Prednisone
PVDF: Polyvinylidene fluoride
PKM2: Pyruvate kinase M2
TLR4: Toll-like receptor 4
SKN: Shikonin
TNF-α: Tumor necrosis factor-α
UA: Uric acid
