# Supplementary material for: Celastrol mitigates inflammation in sepsis by inhibiting the PKM2-dependent Warburg effect
Source: Mil Med Res. 2022 May 20;9:22. doi: 10.1186/s40779-022-00381-4 (PMC9121578; doi:10.1186/s40779-022-00381-4)
Supplement: Supplementary file 1 — Additional file 1: Fig. S1. Chemical structure of celastrol (Cel) and scheme of animal experiments. Fig. S2. Overall workflow of activity-based protein profiling for identifying potential targets of celastrol (Cel). Fig. S3. Celastrol (Cel) protects mice from experimental sepsis and endotoxic shock. Fig. S4. Celastrol (Cel) inhibits the Warburg effect in LPS-induced macrophages. Fig. S5. Celastrol (Cel) binds to Cys residues of PKM1. Fig. S6. Celastrol (Cel) binds to Cys residues of LDHA and inhibits its activity. [file 40779_2022_381_MOESM1_ESM.pdf]

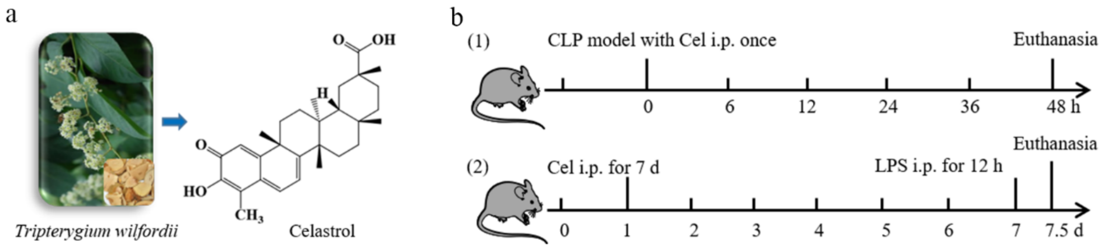

Fig. S1 Chemical structure of celastrol (Cel) and scheme of animal experiments. **a** The *Tripterygium wilfordii* plant and the chemical structure of Cel. **b** Scheme of experiments involving mouse models of experimental endotoxemia and sepsis. CLP cecal ligation puncture, LPS lipopolysaccharide

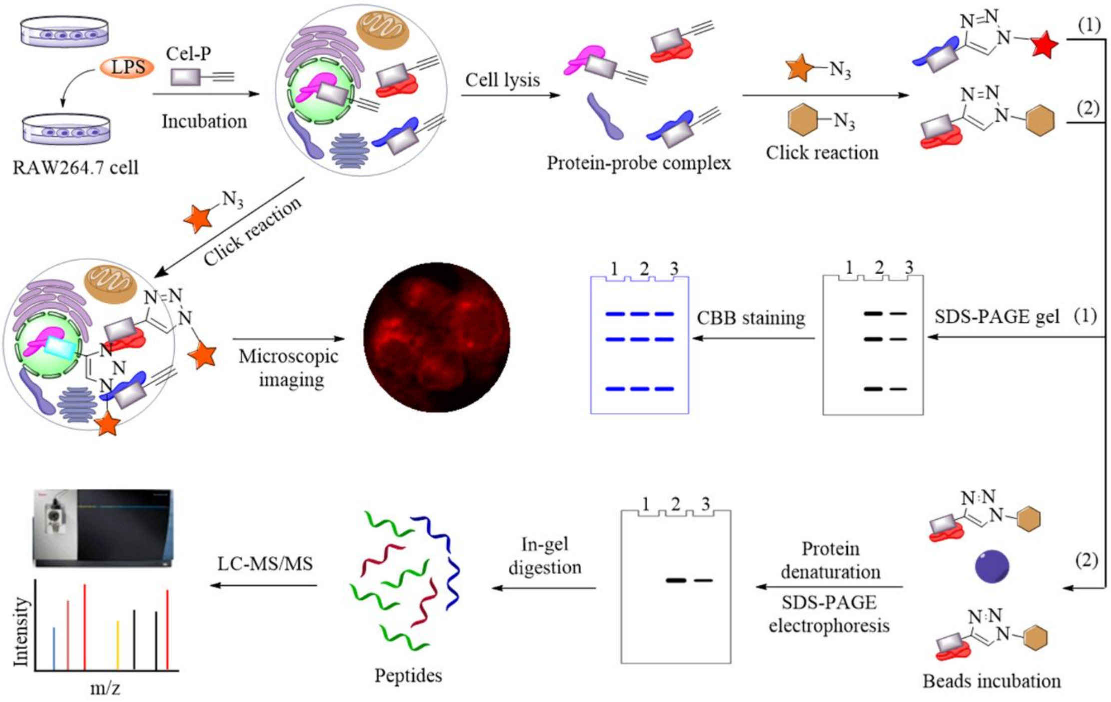

Fig. S2 Overall workflow of activity-based protein profiling for identifying potential targets of celastrol (Cel). LPS lipopolysaccharide, Cel-P celastrol-probe, CBB coomassie brilliant blue, LC-MS/MS liquid chromatography - tandem mass spectrometry

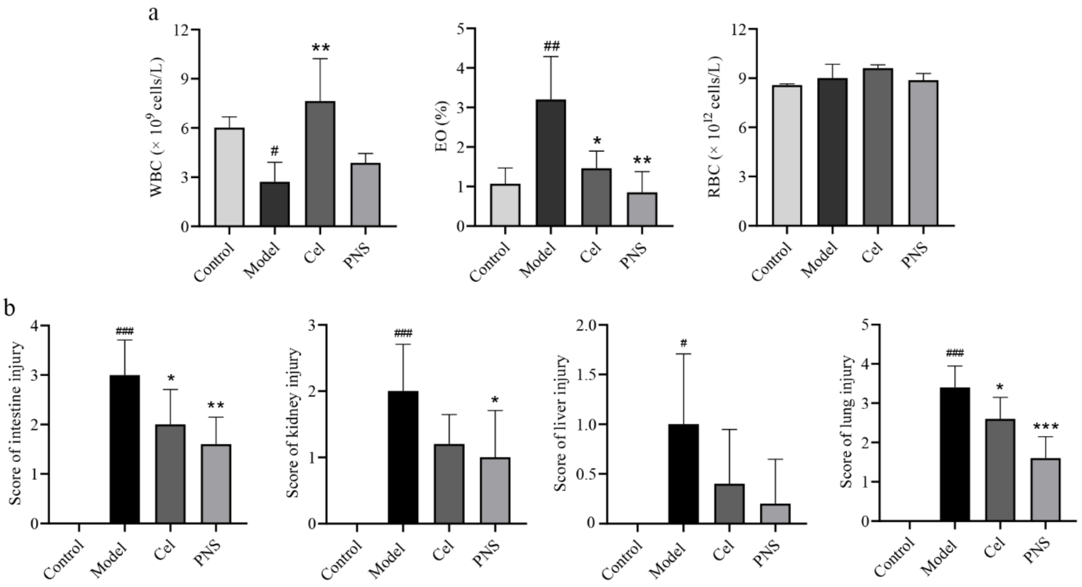

Fig. S3 Celastrol (Cel) protects mice from experimental sepsis and endotoxic shock. **a** Blood cell counts in endotoxemic mice. **b** Histopathology scores for intestine, kidney, liver and lung in mice. All data are expressed as mean ± SEM, n = 3 - 5, $^{\#}P < 0.05$, $^{\#\#\#}P < 0.001$ vs. Control; $^{*}P < 0.05$, $^{**}P < 0.01$, $^{***}P < 0.001$ vs. Model. WBC white blood cell, EO eosinophil, RBC red blood cell, PNS prednisone

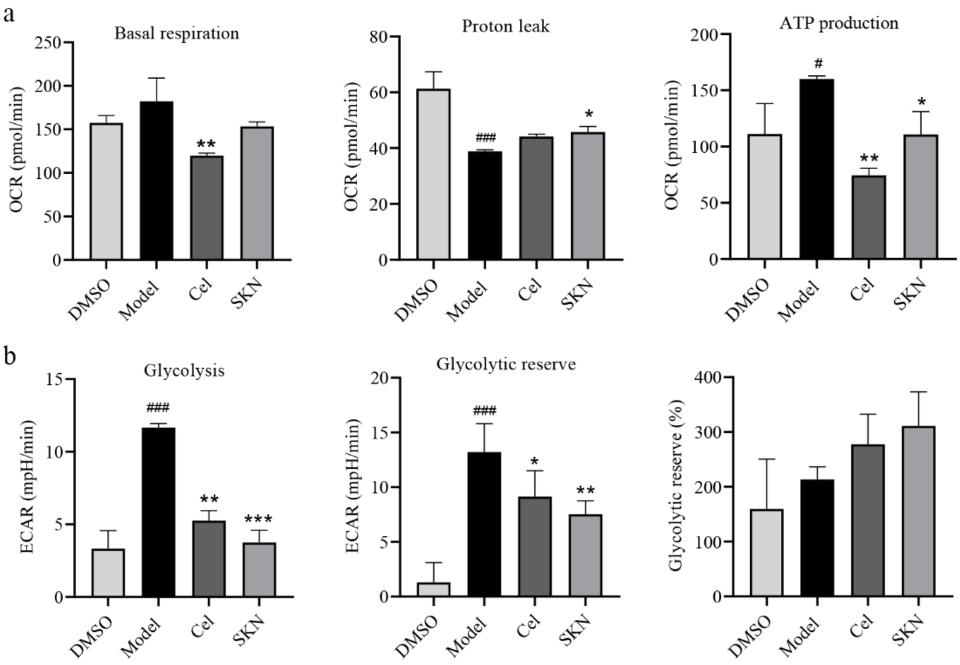

Fig. S4 Celastrol (Cel) inhibits the Warburg effect in LPS-induced macrophages. The grouping as follows: DMSO, Model (LPS 100 ng/ml), Cel (LPS 100 ng/ml + Cel 1 μmol/L), SKN (LPS 100 ng/ml + shikonin 1 μmol/L). **a** Oxygen consumption rate (OCR), as expressed in terms of basal respiration, proton leak and ATP production. **b** Extracellular acidification rate (ECAR), as expressed in terms of glycolysis, absolute glycolytic reserve and glycolytic reserve as a percentage. All data are expressed as mean ± SEM (n = 3). $^{\#}P < 0.05$, $^{\#\#\#}P < 0.001$ vs. DMSO; $^{*}P < 0.05$, $^{**}P < 0.01$, $^{***}P < 0.001$ vs. Model. LPS lipopolysaccharide, SKN shikonin

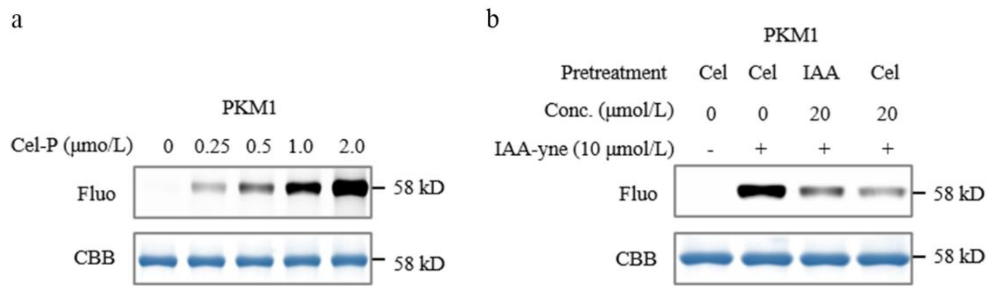

Fig. S5 Celastrol (Cel) binds to Cys residues of PKM1. **a** Recombinant human PKM1 labeled by celastrol-probe (Cel-P) in a dose-dependent manner. **b** Recombinant human PKM1 labeled by alkyne-tagged IAA in the presence or absence of the competitors Cel and IAA. PKM1 pyruvate kinase M1, Fluo fluorescence, CBB coomassie brilliant blue, Conc. concentration, IAA iodoacetamide

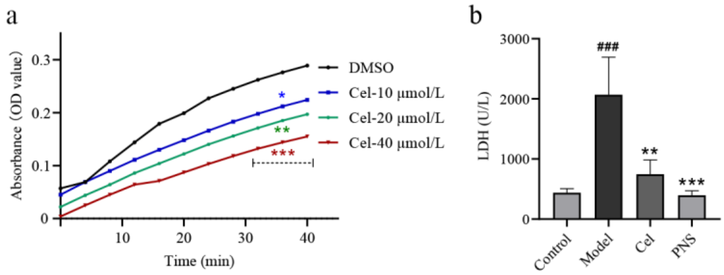

Fig. S6 Celastrol (Cel) binds to Cys residues of LDHA and inhibits its activity. **a** Catalytic activity of LDH in the presence or absence of Cel at 10, 20 and 40 μmol/L. **b** Serum levels of LDH in endotoxemic mice. All data are expressed as mean ± SEM (n = 3). $^{###}P < 0.001$ vs. Control; $^{**}P < 0.01$, $^{***}P < 0.001$ vs. Model. LDH lactate dehydrogenase, PNS prednisone
