# Supplementary material for: Celastrol mitigates inflammation in sepsis by inhibiting the PKM2-dependent Warburg effect
Source: Mil Med Res. 2022 May 20;9:22. doi: 10.1186/s40779-022-00381-4 (PMC9121578; doi:10.1186/s40779-022-00381-4)
Supplement: Supplementary file 2 — Additional file 2: Table S1. Short interfering RNA sequences used to knockdown the PKM2 gene in macrophages. [file 40779_2022_381_MOESM2_ESM.pdf]

Table S1 Short interfering RNA sequences used to knockdown the *PKM2* gene in macrophages

Name	Sense (5' - 3')	Antisense (5' - 3')
si-*PKM2*-1	GACAUGGUGUUUGCAUCUUUCTT	GAAAGAUGCAAACACCAUGUCTT
si-*PKM2*-2	GAUGUCGACCUUCGUGUAAACTT	GUUUACACGAAGGUCGACAUCTT
si-*PKM2*-3	CCAUGCAGAGACCAUCAAGAATT	UUCUUGAUGGUCUCUGCAUGGTT
si-NC	UUCUUCGAACGUGUCACGUTT	ACGUGACACGUUCGGAGAATT

PKM2 pyruvate kinase M2, *NC* negative control
